# Who Benefits the Most from Sleep Hygiene Education? Findings from the SLeep Education for Everyone Program (SLEEP)

**DOI:** 10.3390/clockssleep7030040

**Published:** 2025-08-01

**Authors:** Alyssa Tisdale, Nahyun Kim, Dawn A. Contreras, Elizabeth Williams, Robin M. Tucker

**Affiliations:** 1 Department of Food Science and Human Nutrition, Michigan State University, East Lansing, MI 48824, USA; tisdal14@msu.edu (A.T.); nahyunkim@korea.ac.kr (N.K.); 2 Institute for Physical Activity and Health Promotion, Korea University, Seoul 02841, Republic of Korea; 3 Michigan State University Extension, East Lansing, MI 48824, USA; josaitis@msu.edu

**Keywords:** sleep education, sleep hygiene, sleep health, insomnia, sleep predictors

## Abstract

This study examined data from participants who completed the SLeep Education for Everyone Program (SLEEP) to explore how various demographic variables affected sleep outcomes and to determine which participant characteristics predicted success. A total of 104 individuals participated. The Sleep Hygiene Index (SHI) measured undesirable sleep behaviors; the Pittsburgh Sleep Quality Index (PSQI) assessed sleep quality and self-reported sleep duration. Participant demographic information was collected at baseline. A mixed ANOVA evaluated group differences, and a multiple linear regression model identified predictors of sleep improvements. Change in SHI scores from pre- to post-intervention demonstrated a significant time × group interaction between Black and white participants (*p* = 0.024); further analysis indicated Black participants improved more. Better baseline scores predicted more favorable post-intervention outcomes for SHI, PSQI, and sleep duration. Fewer chronic conditions predicted better post-intervention SHI and PSQI scores. Older age also predicted better SHI scores. More favorable initial scores, fewer chronic conditions, and older age were the strongest predictors of positive outcomes following SLEEP. Improved sleep hygiene, sleep quality, and sleep duration were observed over time within subjects across all groups. In summary, SLEEP appears to be effective. Further work exploring challenges experienced by younger participants or those with multiple co-morbidities is warranted.

## 1. Introduction

Adults in the United States suffer from poor sleep, with one in three sleeping less than the recommended 7 h per night [1]. Along with hours slept, sleep quality, defined as an individual’s satisfaction with their sleeping experience [2], is critical to health [3]. Whether in terms of duration or quality, sleep disturbances are associated with the presence of depression, anxiety, and impaired physical health [4,5]. As sleep problems become increasingly prevalent [6], it is essential to understand possible contributing factors.

Non-pharmacological therapies for improving sleep have been shown to be effective [7,8]. The most studied of these approaches is Cognitive Behavioral Therapy for Insomnia (CBT-I) [9,10,11]. However, CBT-I is not often offered to those with subclinical issues or those without a diagnosable disorder due to a shortage of trained practitioners [12]. Public health initiatives often have greater accessibility; however, they mostly target diet and exercise and ignore sleep [13,14]. Given these challenges, there is a strong need to identify and evaluate effective programs to address subclinical sleep issues for the general public.

The SLeep Education for Everyone Program (SLEEP) is one such program. SLEEP is a non-expert-delivered sleep education program for promoting healthy sleep behaviors. SLEEP utilizes many CBT-I techniques, including Stimulus Control Therapy, relaxation, and sleep hygiene education. The program consists of six group sessions and has been shown to be effective in improving sleep hygiene behaviors and sleep quality [15]. These benefits persisted for at least six months after completing the program [16]. Since 2022, the program has been available free of charge through Michigan State University Extension.

The current study explored the effectiveness of the program within different demographic groups, along with determining which baseline characteristics best predicted favorable outcomes. While prior research has demonstrated the efficacy of similar programs [17,18], few have examined predictors of success. Findings from this study will allow researchers to build a profile of those who are most likely to benefit from the program, or programs like it, and guide potential program modifications to maximize success for those who experience greater challenges.

## 2. Results

A total of 104 adults completed the program between January 2022 and July 2024. Table 1 displays the baseline characteristics of the participants. The population was predominantly white, >65 years old, and female, with an average body mass index (BMI) consistent with an overweight population. The median income for participants was USD 64,313 ± USD 27,236 IQR. At baseline, 91% of participants reported poor sleep quality with PSQI scores ≥ 5, and 64% did not meet the duration guidelines of >7 h of sleep per night [19]. A majority of participants (56%) completed all six sessions.

### 2.1. Sleep Outcomes

**Figure 1:**
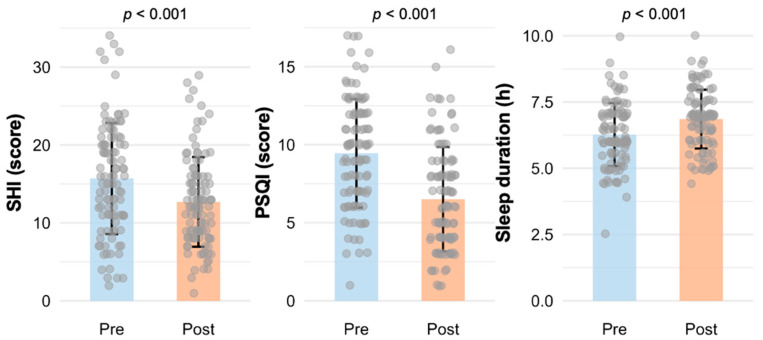
Improvement in sleep parameters after SLEEP. The bar plot represents the pre- and post-intervention mean and standard deviation, with the gray dots indicating individual participant values. The SHI (Sleep Hygiene Index) measures the frequency of engaging in behaviors that can impair sleep; lower scores are more favorable. The PSQI (Pittsburgh Sleep Quality Index) assesses sleep quality. Self-reported duration was extracted from the PSQI. Missing data: SHI (*n* = 2); PSQI (*n* = 3); sleep duration (*n* = 2).

Participants reported less frequent engagement in undesirable sleep hygiene behaviors (SHI scores) after completing the program (15.7 ± 7.1 vs. 12.7 ± 5.8; *p* < 0.001). PSQI scores, reflecting sleep quality, improved post-intervention (9.5 ± 3.5 vs. 6.5 ± 3.4; *p* < 0.001). Self-reported sleep duration, derived from the PSQI, increased by half an hour (6.3 ± 1.2 (h) vs. 6.8 ± 1.1 (h); *p* < 0.001). To further evaluate individual-level improvements, the percentage change in sleep duration was calculated. On average, sleep duration increased by 11.5% (SD = 20.3%), and 48% of participants (49 out of 102) exhibited a ≥10% increase. These findings suggest that the intervention produced notable improvements for a substantial portion of participants. The outcomes are displayed in Figure 1.

### 2.2. Differences in Outcomes

**Figure 2:**
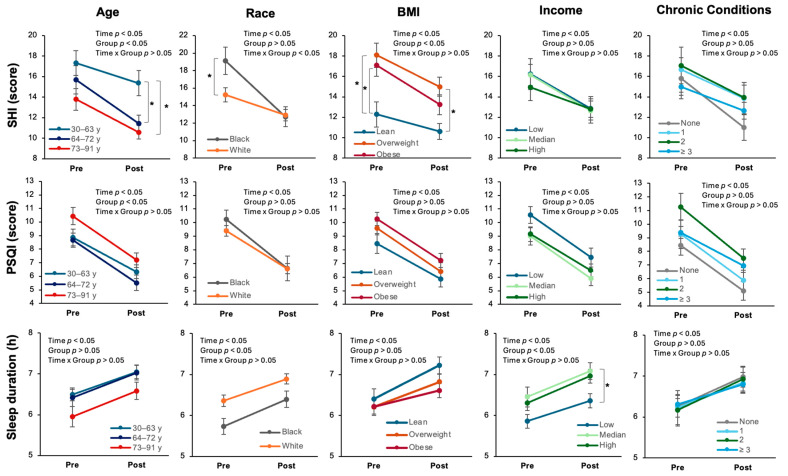
Effects of SLEEP on sleep outcomes across groups defined by age, race, BMI, income, and chronic conditions. * *p* < 0.05.

Participants were grouped by age, race, BMI category, income status, and number of chronic conditions to determine differences in sleep outcomes based on social and economic characteristics. Figure 2 illustrates the ANOVA analyses for sleep outcomes by time, group, and time × group interactions. Significant main effects of time were observed in SHI, PSQI, and sleep duration from pre- to post-intervention across all groups (*p* < 0.001), indicating that all sleep outcomes improved over time, regardless of group classification.

#### 2.2.1. Age

Age groups were categorized based on tertiles of the sample population. Time × group interactions were not detected for any sleep outcome. For SHI scores, a significant main effect of age group was observed (*p* = 0.006). Further analysis indicated that participants in the youngest tertile (30–63 y) had significantly higher (more undesirable) post-intervention SHI scores compared to both the middle (64–72 y; *p* = 0.007) and oldest tertile (73–91 y; *p* = 0.002). For PSQI scores, a significant main effect of age group was observed (*p* = 0.027); however, further analysis did not indicate significant group differences at either time point. No significant group differences were observed for sleep duration. Linear mixed model analyses (Table 2), adjusted for socioeconomic covariates, were conducted to examine associations between demographic variables (age, race, BMI, income, and number of chronic conditions) and the three sleep outcomes while controlling for education level, employment status, and median income. After adjusting for SES variables in age, all sleep outcomes sustained improvement over time, with no significant time × group interaction.

#### 2.2.2. Race

Analyses based on race were conducted using data from people who identified as Black (*n* = 19) or white (*n* = 80). Significant time × group interactions were observed for the SHI scores (*p* = 0.024), identifying differences between races over time. Black participants had higher (more undesirable) SHI scores at baseline compared to white participants (*p* = 0.036); however, post-program scores of Black participants were equal to those of white participants, suggesting that Black participants experienced a greater reduction in adverse sleep hygiene behaviors. Neither the PSQI nor sleep duration analysis detected significant time × group interactions. There were no significant group differences in PSQI scores at either time point. For sleep duration, a significant main effect for race was discovered; however, differences between groups by time were not observed. Table 2 displays the linear mixed model analyses based on race, controlling for socioeconomic factors. The significant time × group interaction remained for SHI scores (*p* = 0.020) after controlling for SES covariates. Specifically, Black participants experienced a greater improvement in SHI over time compared to white participants, regardless of education, employment, or income differences. No such time × group interaction was observed for the PSQI or sleep duration.

#### 2.2.3. BMI

Participants were grouped by BMI category (lean, overweight, and obese). Time × group interactions were not observed for any sleep outcome. A significant main effect by BMI group was observed for SHI scores. Further analysis determined that individuals classified as lean reported better scores pre-intervention compared to individuals classified as overweight (*p* = 0.006) or obese (*p* = 0.010). The post-intervention SHI scores remained better among lean individuals compared to those in the overweight category (*p* = 0.012); however, differences between the lean and obese groups were no longer observed. The PSQI and sleep duration analyses did not indicate group differences at either time point. In the linear mixed model analyses of BMI, no significant time × group interaction for any of the sleep outcomes after adjusting for SES variables were observed (Table 2).

#### 2.2.4. Income

Participants were grouped by income based on tertiles of the sample. No time × group interactions were detected in the three sleep outcomes. There were no significant group differences in SHI and PSQI scores at either time point. Sleep duration analysis discovered a significant main effect by income group (*p* = 0.033). Further analysis indicated that middle-income individuals (USD 53,045–80,280) reported significantly longer sleep duration post-program compared to participants in the lower-income group (<USD 53,045; *p* = 0.020); the durations for middle- and high-income individuals did not differ at either the pre- or post-program time points. Table 2 displays the linear mixed model analyses, adjusted for socioeconomic covariates, for the income group; median income was excluded in the income group analysis, as it served as the grouping variable. After adjusting for SES variables, no significant time × group interaction was found across any sleep outcomes.

#### 2.2.5. Chronic Conditions

No significant time × group interactions were revealed for the SHI, PSQI, or sleep duration. Additionally, there were no significant group differences for the SHI, PSQI, or sleep duration at either time point. After adjusting for SES covariates, overall improvements over time remained, and no significant time × group interactions were noted (Table 2).

### 2.3. Sleep Improvement Predictors

Results from the multiple regression analyses identified models that best predicted post-program outcomes (Table 3). Older age, fewer chronic conditions, and more favorable initial SHI scores predicted a lower, more favorable SHI score post-program (adjusted R^2^ = 0.35; df = 83; F = 15.72; *p* < 0.001). Having fewer chronic conditions and reporting better sleep quality at baseline predicted a lower, more favorable PSQI score post-intervention (adjusted R^2^ = 0.28; df = 82; F = 16.54; *p* < 0.001). Longer baseline sleep duration predicted longer sleep duration post-intervention (adjusted R^2^ = 0.36; df = 83; F = 47.94; *p* < 0.001).

## 3. Discussion

For each of the three sleep measures (sleep hygiene, sleep quality, and sleep duration), more favorable baseline scores predicted more favorable post-program scores. Additionally, fewer chronic conditions predicted better post-intervention sleep hygiene and sleep quality scores. Older age also emerged as a predictor of favorable sleep hygiene scores following the intervention. In contrast, race, education level, employment, BMI, and income did not significantly predict post-intervention outcomes, suggesting that these variables had minimal impacts on sleep improvements following SLEEP.

Better baseline scores predicted better post-program sleep hygiene, sleep quality, and sleep duration. Thus, it appears those with healthier sleep habits prior to enrolling in SLEEP more easily furthered their healthy behaviors. Conversely, less favorable baseline scores may indicate profound sleep issues (e.g., insomnia disorder, sleep apnea, restless leg syndrome), which might not be adequately addressed through behavior change alone. Therefore, these individuals would likely benefit from a comprehensive sleep study to identify diagnosable conditions and determine further treatment options.

Chronic conditions emerged as a significant predictor in post-program sleep hygiene and sleep quality, suggesting that those with a higher number of chronic conditions tended to report less desirable sleep hygiene and sleep quality post-program. However, no differences between subjects and within subjects were found. Those with multiple chronic conditions might require a higher level of sleep care, as changes in sleep hygiene behaviors may not address the root cause of their sleep issues. Treatment of chronic disease, specifically multimorbidity, is often associated with reduced quality of life, increased emotional stress, and decreased ability to care for oneself [20,21,22,23]; these adverse effects may cause greater sleep problems compared to adults with fewer diseases [24]. Additionally, individuals with chronic conditions are more likely to take multiple medications [25], making drug side effects and drug-to-drug interactions more prevalent [26]. Many of these medications can induce or exacerbate sleep issues [27,28]. Thus, individuals with multiple chronic conditions may benefit from further sleep-related care and discussion with their medical provider about drug-related side effects impacting sleep.

Age was positively associated with better sleep hygiene habits, and older adults experienced greater improvements in sleep hygiene behaviors post-program compared to younger participants. While aging is commonly assumed to cause sleep problems, multiple studies indicate that sleep issues in older adults are more likely due to increased health complications rather than aging itself [29,30,31]. Older adults are more likely to prioritize their sleep, with generally better attitudes towards the benefits of sleep and more autonomy in their daily routines [32,33]. These factors may explain why older age predicted better sleep hygiene post-program, as well as why older adults experienced greater improvement when compared to younger adults.

Health disparities, which often result from a combination of social, economic, and environmental factors, can be detrimental to achieving one’s highest level of health [34], specifically, sleep health [35,36,37]. Poor access to sleep education is a key component to continued disparity [38]; this limited access is more prominent among individuals who are Black [39]. In the United States, Black Americans often experience less optimal sleep duration and quality [40,41]; a focus group on sleep perceptions in Black individuals attributed suboptimal sleep to stress, responsibilities, and sleep-hindering behaviors [42]. Comparably, results from the current study indicated that Black individuals reported more undesirable sleep hygiene habits pre-program than white individuals. However, these differences were no longer present after SLEEP, suggesting Black individuals experienced greater improvement in sleep hygiene behaviors. The observed differences between the two groups remained significant after adjusting for socioeconomic variables, indicating that these differences cannot be accounted for by education, employment status, or income. Sleep duration was higher pre- and post-intervention in individuals who were white compared to individuals who were Black, although both experienced improvements over time. These results suggest that SLEEP can improve outcomes in both Black and white participants; however, Black individuals may experience greater benefit, even with a less desirable initial sleep hygiene score or lower sleep duration at baseline. These marked improvements by Black participants likely explain why race was not a predictor of post-program outcomes.

Another factor contributing to health disparities is income level. Income status directly impacts health care access due to fewer resources and opportunities [43]. In a recent systematic review on socioeconomic status and sleep, higher income was associated with better sleep efficiency and longer sleep duration [37]. In this study, participants in the lowest income range experienced shorter sleep duration post-program compared to those in the middle-income bracket; however, income did not predict post-intervention outcomes, as both groups experienced more favorable outcomes post-intervention. Further, the SHI and PSQI scores at baseline and post-intervention did not differ among income groups. While health disparities are known to interfere with health and education access [35,44,45], SLEEP emerges as an overall impactful education program for all, regardless of economic factors.

There is an inverse relationship between sleep problems and weight status, with short sleep duration being strongly associated with higher weight [46,47]. In contrast, in the present study, BMI classification did not predict post-SLEEP outcomes, suggesting that adiposity did not impact the overall outcomes, including sleep duration. Further analysis observed sleep hygiene differences among BMI categories. Individuals who were classified as overweight engaged in more unfavorable sleep hygiene practices at both baseline and post-program compared to lean individuals. Similarly, a difference also occurred pre-program between the lean and obese groups; however, these differences were not present post-program. These findings suggest that although individuals with obesity had poorer baseline scores, the post-program outcomes were comparable between the two groups. Regression analysis indicated that BMI status did not predict post-SLEEP outcomes. Therefore, the mixed ANOVA results indicate that individuals with higher BMIs may initially exhibit poorer sleep hygiene; however, SLEEP appears equally beneficial across BMI groups when other contributing factors are considered.

The results of this single-arm sleep education intervention revealed differences among sub-groups and predictors of sleep outcomes following SLEEP. The use of regression analysis coupled with mixed ANOVA provided a better understanding of group differences and the relationships influencing the overall outcomes. Although some missing data occurred during the regression analysis, the final analytic sample size (83–84 participants) was sufficient to ensure adequate statistical power, particularly given that the regression models included only one to three predictor variables. Little’s MCAR test indicated that the missing data were missing completely at random, suggesting that the use of listwise deletion was statistically justifiable and unlikely to have introduced systematic bias. Undiagnosed sleep disorders may have been present in the population prior to undergoing the program, influencing the overall outcomes. Limitations in generalizability are present due to the sex and age composition of the sample population, with only ten male participants and an average age of >66 years. Limitations in generalizability are also present in terms of race, as this study had a total of 19 Black participants; however, post hoc testing ensured that the sample size was sufficient for the analyses used. Sample income data were obtained based on zip code to avoid sensitive questioning; this may have affected income-related results. Results were based on subjective measures of sleep only; objective measures should be utilized in the future to corroborate subjective findings.

## 4. Materials and Methods

As mentioned above, the SLeep Education for Everyone Program (SLEEP) is a 6-week program consisting of 30 min group sessions, delivered weekly. Topics include sleep hygiene behavior, Stimulus Control Therapy, mindfulness practices to establish awareness of the present moment, physical activity’s effect on sleep, and exposure to sleep myths and truths for sleep improvement. Each session features a brief educational video, followed by group discussion and individual goal setting. The educational videos offer behavior change recommendations to improve sleep health, and participants select a behavior change goal to incorporate each week that is relevant to their lifestyle.

This study used a single-arm intervention design to explore the predictors of sleep outcomes after completion of SLEEP. The sample population consisted of those who voluntarily enrolled in the program through Michigan State University Extension between January 2022 and July 2024. Trained MSU Extension health educators facilitated the sessions. Participants chose to participate in an in-person or virtual program, as previous work demonstrated equivalent outcomes in delivery modalities [48]. Participants gave consent prior to enrollment. The MSU Human Research Protection Program approved the study (STUDY00005756).

### 4.1. Participants

SLEEP was advertised through the Extension website and social media. The targeted population included adults who were at least 18 years of age and dissatisfied with their sleep duration and/or quality. Demographic characteristics were assessed at baseline, including age, gender, race, ethnicity, education level, employment status, zip code, and number of people living in the home.

### 4.2. Measures

Subjective sleep outcomes based on standardized questionnaires were measured at baseline and immediately following the intervention. The main outcomes were sleep quality, sleep duration, and sleep hygiene.

#### 4.2.1. Sleep Hygiene Index (SHI)

The frequency of engaging in undesirable sleep hygiene behaviors was measured using the Sleep Hygiene Index (SHI). The 13-item questionnaire investigates behaviors like inconsistent bed and wake times and watching TV in bed [49]. Each item is rated on a five-point Likert scale (never: 0; rarely: 1; sometimes: 2; frequently: 3; always: 4). The total scores are summed from 0 to 52, with higher scores being less favorable and indicating poorer sleep hygiene habits.

#### 4.2.2. Pittsburgh Sleep Quality Index (PSQI)

Sleep quality and duration were measured using the Pittsburgh Sleep Quality Index (PSQI), a validated 19-item questionnaire [50]. The 19 items generate seven domain scores: subjective sleep quality, sleep latency, sleep duration, sleep efficiency, sleep disturbance, sleep medication, and daytime dysfunction. The summed domain scores produce a global score ranging from 0 to 21; a score of >5 implies poor sleep quality. Self-reported sleep duration (h) was extracted from the PSQI.

### 4.3. Other Measures

Demographic and health information was self-reported. Information on race was obtained due to well-documented sleep and health disparities in the literature.

Income levels were estimated using the 5-year 2022 American Community Survey (ACS) for each participant based on self-reported zip codes. Zip codes were used as a proxy for income to avoid asking sensitive questions.

Body mass index (BMI) was calculated from the participant’s self-reported height and weight. BMI values were classified into three categories: lean (>18.5–24.9 kg/m^2^), overweight (25.0–29.9 kg/m^2^), and obese (>30.0 kg/m^2^).

### 4.4. Statistical Analysis

A mixed ANOVA was conducted to examine the differences in sleep education outcomes before and after the intervention across three BMI categories (lean, overweight, obese), age (grouped into tertiles based on the sample population), number of chronic conditions (grouped into one, two, and three or more), race (grouped based on self-identification as Black or white), and income categories (low, medium, or high, determined by tertiles of the sample population). One-way ANOVA was performed separately to assess group differences at each time point (pre- and post-intervention). Levene’s test for equality of variances was conducted prior to ANOVA to confirm the homogeneity of variance across groups. Main and interaction effects were evaluated using F-statistics and *p*-values. Bonferroni correction was applied for post hoc comparisons when significant effects were detected. Normality was assessed using the Shapiro–Wilk test and Q-Q plots. Linear mixed model (LMM) analyses were conducted to examine the associations between demographic and social variables (age, race, BMI, income, and chronic conditions) and sleep outcomes (SHI, PSQI, and sleep duration), while controlling for socioeconomic covariates, including education level, employment status, and median income by ZIP code. Fixed effects included time, group, and the time × group interaction. Participant ID was included as a random effect to account for repeated measures within individuals. For each model, estimated coefficients (β), standard errors (SEs), 95% confidence intervals (CIs), and *p*-values were reported. Linear mixed models were estimated using restricted maximum likelihood. This approach accommodates missing outcome data under the assumption of missing at random (MAR), without excluding participants listwise. Multiple linear regression analyses were conducted to examine factors associated with improved post-program sleep hygiene practices, sleep quality, and sleep duration. Pre-program scores were included as covariates to adjust for baseline differences, along with demographic variables. The stepwise selection method was applied to determine the most relevant independent variables contributing to changes in primary outcome scores. Regression analyses were performed using listwise deletion, whereby participants with missing data on any covariates were excluded from the analysis. As a result, the final sample size ranged from 83 to 84, depending on the specific model. Little’s MCAR test was conducted to confirm that the missing data were missing completely at random (MCAR) prior to proceeding with the analysis. Independence of residuals was tested using the Durbin–Watson test to ensure the validity of the regression model. To assess model performance, adjusted R^2^ was examined to evaluate the model’s explanatory power, while *p*-values were used to determine statistical significance. The variance inflation factor (VIF) was checked to identify potential multicollinearity issues among predictors. A priori power analysis was conducted using G*Power (Version 3.1, Heinrich-Heine-Universität Düsseldorf, German) to estimate the required sample size for multiple linear regression, assuming a medium effect size (f^2^ = 0.15), α = 0.05, and power = 0.80. The analysis indicated that a minimum of 77 participants was sufficient to detect significant effects in a model with three predictors. Among the 104 participants, complete data were available for 83 to 84 individuals across the regression models. Post hoc power analyses using the same parameters confirmed that all models achieved sufficient statistical power. The achieved power ranged from 0.84 to 0.93 across the three regression models. Statistical analyses were performed using SPSS (Version 21, IBM Corp., Armonk, NY, USA) and R (Version 4.4.0), and a two-sided *p* < 0.05 was considered statistically significant. Data are represented as mean ± standard deviation (SD).

## 5. Conclusions

Better initial sleep hygiene, quality, and duration measures predicted better sleep outcomes for their respective post-program measures. Fewer chronic conditions predicted more favorable SHI and PSQI post-program scores. Older age predicted a better SHI post-program score. Race, education level, employment, BMI status, and income range had negligible influence on predicting sleep improvements following SLEEP. Improved sleep hygiene, sleep quality, and sleep duration were observed over time within all subject groups analyzed: age, race, BMI, income, and number of chronic conditions. In summary, SLEEP appears to be effective for a large segment of the population; however, further work exploring the challenges experienced by younger participants or those with multiple co-morbidities is warranted.

## Figures and Tables

**Table 1:** Characteristics of participants enrolled in the SLEEP intervention.

	n (%)	Mean (SD)
Age		66.7 y (12.3)
- Reported - Undisclosed	99 (95.2%) 5 (4.8%)	
Sex		
- Female - Male - Undisclosed	93 (89.4%) 10 (9.6%) 1 (1.0%)	--
Race		
- White - Black - Asian - Undisclosed	80 (76.9%) 19 (18.3%) 3 (2.9%) 2 (1.9%)	--
BMI		29.9 kg/m^2^ (8.1)
- Underweight (<18.5 kg/m^2^) - Lean (≥18.5–24.9 kg/m^2^) - Overweight (25.0–29.9 kg/m^2^) - Obese (≥30.0 kg/m^2^) - Undisclosed	0 (0%) 31 (29.8%) 25 (24.0%) 45 (43.3%) 3 (2.9%)	
Number of Chronic Diseases		
- 0 - 1 - 2 - ≥3	21 (20.2%) 15 (14.4%) 16 (15.4%) 52 (50.0%)	--
Median Income in Dollars ^1^		
- Low (<USD 53,045) - Medium (USD 53,045–80,280) - High (≥USD 80,281) - Undisclosed	25 (24.0%) 52 (50.0%) 25 (24.0%) 2 (1.9%)	--

Data presented as n (%) and/or mean (SD). ^1^ Median income data based on zip code were obtained from the 2022 American Community Survey 5-Year estimates. Low-, medium-, and high-income ranges were determined based on the 25th and 75th percentiles of the study population.

**Table 2:** Associations between group variables and sleep outcomes, controlled for socioeconomic covariates, in linear mixed model analyses.

Outcome	Group Variable	Effect	Estimate (β)	SE	95% CI		*p*-Value
					Lower	Upper	
SHI							
	Age	Time	−2.38	1.91	−6.17	1.41	<0.001 **
		Group	−1.14	1.50	−4.11	1.82	0.028 *
		Time × Group	−0.42	0.89	−2.18	1.34	0.429
	Race	Time	−14.46	4.93	−24.26	−4.65	0.003 *
		Group	−8.08	3.03	−14.07	−2.09	0.173
		Time × Group	4.05	1.74	0.58	7.51	0.020 *
	BMI	Time	0.68	2.81	−4.90	6.27	0.809
		Group	3.20	1.45	0.33	6.07	0.032 *
		Time × Group	−1.19	0.86	−2.88	0.51	0.165
	Income	Time	−4.25	2.14	−8.50	0.00	0.047 *
		Group	−1.05	1.7	−4.42	2.32	0.837
		Time × Group	0.59	1.00	−1.40	2.58	0.555
	Chronic Conditions	Time	−4.75	1.34	−7.41	−2.09	<0.001 **
		Group	−1.36	1.00	−3.33	0.61	0.888
		Time × Group	0.86	0.58	−0.30	2.02	0.140
PSQI							
	Age	Time	−2.23	0.90	−4.02	−0.45	<0.001 **
		Group	1.42	0.73	−0.02	2.86	0.045 *
		Time × Group	−0.42	0.42	−1.24	0.41	0.616
	Race	Time	−5.37	2.40	−10.14	−0.61	0.025 *
		Group	−1.45	1.53	−4.47	1.58	0.715
		Time × Group	0.83	0.85	−0.85	2.52	0.325
	BMI	Time	−2.36	1.34	−5.02	0.30	0.077
		Group	0.94	0.72	−0.48	2.36	0.091
		Time × Group	−0.21	0.41	−1.02	0.60	0.600
	Income	Time	−3.65	1.02	−5.68	−1.63	<0.001 **
		Group	−0.76	0.84	−2.42	0.90	0.498
		Time × Group	0.31	0.48	−0.64	1.25	0.521
	Chronic Conditions	Time	−3.54	0.64	−4.82	−2.27	<0.001 **
		Group	0.01	0.49	−0.98	0.97	0.132
		Time × Group	0.26	0.28	−0.30	0.82	0.357
Sleep Duration							
	Age	Time	0.49	0.29	−0.09	1.07	<0.001 **
		Group	−0.37	0.24	−0.85	0.11	0.109
		Time × Group	0.06	0.13	−0.21	0.33	0.870
	Race	Time	0.94	0.74	−0.54	2.41	0.206
		Group	0.70	0.48	−0.26	1.65	0.072
		Time × Group	−0.13	0.26	−0.65	0.39	0.615
	BMI	Time	0.62	0.11	0.41	0.84	<0.001 **
		Group	0.19	0.23	−0.27	0.65	0.174
		Time × Group	−0.24	0.13	−0.49	0.01	0.052
	Income	Time	0.42	0.32	−0.22	1.07	0.188
		Group	0.06	0.27	−0.48	0.59	0.236
		Time × Group	0.08	0.15	−0.22	0.38	0.582
	Chronic Conditions	Time	0.73	0.20	0.32	1.13	<0.001 **
		Group	0.12	0.16	−0.20	0.44	0.855
		Time × Group	0.07	0.09	−0.24	0.11	0.441

SHI (Sleep Hygiene Index). PSQI (Pittsburgh Sleep Quality Index). Socioeconomic covariates included education level, employment status, and median income by ZIP code; median income was excluded in the income group analysis, as it served as the grouping variable. Estimated coefficients (β); SE = standard errors; 95% CI = confidence intervals. * *p* < 0.05. ** *p* < 0.001.

**Table 3:** Multiple linear regression models for post-intervention sleep outcomes.

Model	B	SE	Beta	*p*
SHI—post				
- Age - Number of chronic conditions - Baseline SHI	−0.177	0.041	−0.407	<0.001 **
	3.309	1.106	0.268	0.004 *
	0.186	0.075	0.235	0.015 *
PSQI—post				
- Number of chronic conditions - Baseline PSQI	0.552	0.251	0.209	0.031 *
	0.427	0.085	0.474	<0.001 **
Sleep Duration—post				
- Baseline duration	0.556	0.080	0.607	<0.001 **

SHI (Sleep Hygiene Index). PSQI (Pittsburgh Sleep Quality Index). B = Unstandardized regression coefficient; SE = standard error of B; Beta = standardized regression coefficient. * *p* < 0.05. ** *p* < 0.001.

## Data Availability

Requests for data can be made to the corresponding author. The data are not publicly available due to ongoing analyses.
